# The prevalence of drug resistance among treatment-naïve HIV-1-infected individuals in China during pre- and post- 2004

**DOI:** 10.1186/s12879-016-1928-x

**Published:** 2016-10-26

**Authors:** Hanping Li, Shuai Chang, Yang Han, Daomin Zhuang, Lin Li, Yongjian Liu, Siyang Liu, Zuoyi Bao, Wenfu Zhang, Hongbin Song, Taisheng Li, Jingyun Li

**Affiliations:** 1 State Key Laboratory of Pathogen and Biosecurity, Institute of Microbiology and Epidemiology, Academy of Military Medical Science, No. 20 East Street, Fengtai district Beijing, 100071 China; 2 Institute of Disease Control and Prevention, Academy of Military Medical Science, Beijing, 100071 China; 3 Department of Clinical Laboratory, PLA Army General Hospital, Beijing, 100700 China; 4 Department of Infectious Disease, Peking Union Medical College Hospital, Peking Union Medical College, Chinese Academy of Medical Sciences, No. 1 Shuaifuyuan Wangfujing, Dongcheng district Beijing, 100730 China

**Keywords:** HIV, Prevalence, Treatment-naïve, Transmitted drug resistance, Antiretroviral therapy

## Abstract

**Background:**

The widespread use of antiretroviral therapies has led to considerable concerns about the prevalence of drug-resistant, as transmission of drug-resistant (TDR) strains poses a challenge for the control of the HIV-1 epidemic.

**Methods:**

We conducted an epidemiological study enrolling treatment-naïve HIV-1-positive subjects at the Peking Union Medical College Hospital since 1991. Drug resistance was determined by submitting the sequences to the Stanford University Network HIV-1 database.

**Results:**

Of 521 participants, 478 samples were amplified and sequenced successfully. HIV Transmitted drug resistance prevalence in China was determined to be 6.7 %. We did not find significant differences in the TDR rate by demographic characteristics. No significant time trend in the prevalence of overall TDR was observed (*p* > 0.05).

**Conclusions:**

We identified an intermediate prevalence of transmitted drug resistance (TDR), exhibiting a stable time trend. These findings enhance our understanding of HIV-1 drug resistance prevalence and time trend, and provide some guidelines for the comprehensive public health strategy of TDR prevention.

## Background

The “Four Frees and One Care” policy implemented in China in 2003, which provided free antiretroviral (ARV) drugs, has been credited for significantly reducing the rate of morbidity and mortality attributed to HIV infection in the country [1, 2]. By August 31st 2013, HIV-1 patients living in 31 provinces and 2215 counties had received antiretroviral therapy (ART) and greatly benefited from this program. The accumulative total of treatment-experienced patients was 234,655, with 188,126 being treated in China.

Characterized by a high replication rate [3] and the error-prone reverse transcriptase [4, 5], HIV-1 is extensively genetically diverse. Like those in developed countries, the emergence and transmission of drug-resistant HIV-1 strains is of considerable concern with the widespread use of ART in developing countries. It has been reported that many treated patients developed resistance to a specific drug within a class or an entire class of ART drugs [6], which may lead to treatment failure and limitation of alteration of treatment regimen. Furthermore, transmitted drug resistance (TDR), which is the transmission of drug-resistant variants to individuals who have never undergone ART can occur. TDR can compromise the efficacy of combination ARV regimens in the management of HIV disease, becoming a major obstacle to ART. The prevalence of TDR varies worldwide ranging from 3.2 % to 24.1 % [2, 7–22]. In China, free therapy was provided to AIDS patients in 2004, which means that there might be transmission of drug-resistant variants. Some reports have demonstrated the increasing TDR rate [22, 23]. So in this study we focused on the compare of the prevalent ratio of drug-resistant variants before and after 2004 in China to provide some guidelines for the TDR prevention.

## Methods

### Patient samples

Plasma specimens were collected from HIV-1 treatment-naïve patients enrolled in the Peking Union Medical College Hospital. Participants had received no antiretroviral drugs at the time of sampling. The patients recruited in this study were AIDS stage for their CD4 cells being below 350 counts per microlitre and viral load exceeding 10^3^ log. Plasma samples were shipped on dry ice and stored at -80 °C until analysis. All patients provided a written informed consent and their basic personal and clinical information was acquired by face to face interview. A total of 521 patients were enrolled, and this study was approved by the Ethics Committee at the Institutional Review Board of the Academy of Military Medical Sciences.

### Genotypic resistance testing

Nested PCR was employed to amplify the 2161 bp HIV-1 gag-pol gene which comprised the entire PR gene (codons 1–99) and RT gene (codons 1–560). HIV-1 RNA was extracted from 500 μl plasma using the QIAamp® Viral RNA Mini Kit (Qiagen) and used as a template for a one-step reverse transcriptase polymerase chain reaction (RT-PCR). The RT-PCR primers were [*MAW26*: 5’-TTGGAAATGTGGAAAGGAAGGAC-3’; *Vif*: 5’-TGACTTTGGGGATTGTAGGGAATA-3’]. The one-step RT-PCR reaction mixture (One Step RNA PCR Kit (AMV), Takara) contained 5 mM MgCl_2_, 1 mM dNTP, 10X One Step RNA PCR Buffer (5 μl), 40 U RNase Inhibitor, 5 U AMV RTase XL, 5 U AMV-optimized Taq, 20 μM (each) primer and 2 μl of viral RNA, for a final volume of 50 μl. Two microliters of the RT-PCR mixture was used for nested PCR, with 1.25 U Premix Ex Taq (Premix Ex Taq^TM^ Version 2.0, Takara) and 20 μM (each) primer [PRO-1: 5’-CAGAGCCAACAGCCCCACCA-3’; RTO-2: 5’-TAAAATCACTAGCCATTGCTCTCC-3’, respectively] combined to a total volume of 50 μl. Both one-step RT-PCR and nested PCR amplifications were carried out in a Biometra Thermocycler (Biometra). The patient-derived PR-RT PCR products were directly sequenced with pyrosequencing method by the Huada Genomics Company (China). The sequences were edited using the ContigExpress software and aligned using the BioEdit program. For each patient, genotypic resistance was determined by submitting the sequences to the Stanford University Network HIV-1 database [24].

### Subtype identification

Subtype was determined by phylogenetic tree analysis of the PR-RT fragments. Alignments also included the reference sequences representing HIV-1 genetic circulating recombinant forms obtained from the HIV Database [25]. The phylogenetic tree was constructed using the Neighbor-Joining method by MEGA (Molecular Evolutionary Genetic Analysis Software, Version 5.4) and genetic distance reliability was evaluated using the bootstrap test (500 bootstrap replicates).

### Statistical analysis

Fisher test analysis was performed using SAS version 9.2. All analyses were performed using a two-tailed test and *p* values less than 0.05 were defined as statistically significant.

## Results

### Patient characteristics

The Peking Union Medical College Hospital provided subjects from a widespread referral network within each of 8 provinces and metropolitan cities across China (Beijing, Shanghai, Guangzhou, Shenzhen, Fuzhou, Henan, Yunnan, and Xi’an) who were treatment-naïve at the time of sampling. A total of 521 subjects infected with HIV-1 during the period 1991–2009 were evaluated; and 491 (94.2 %) of the subjects were from 2005 to 2009. The 28 samples in year 1999–2004 are mainly from Henan (17/28, 60.7 %). Table 1 summarizes demographic and clinical characteristics of the study population. The average age was 35.49 ± 10.01 (18–65 years) and males dominated the study population (69.3 %). More than half (77.9 %) of patients were infected by sexual contact, of which 214 (41.1 %) was through heterosexual contact and 182 (34.9 %) through sex between men. The mean HIV viral RNA load was 4.54 log copies/ml.

**Table 1:** Demographic and clinical characteristics of HIV-1+ patients

Patients, no.	521
Age, mean(min-max)	35.49(18-65)
Sex, no.(%)	
Male	361(69.3)
Female	126(24.2)
Unknown	34(6.5)
Area of origin, no.(%)	
Beijing	112(21.5)
Yunnan	95(18.2)
Shanghai	75(14.4)
Henan	60(11.5)
Fuzhou	52(10.0)
Shenzhen	51(9.8)
Guangzhou	50(9.6)
Xi’an	26(5.0)
Route of transmission, no.(%)	
Heterosexual contact	214(41.1)
Men who have sex with men	182(34.9)
Blood	47(9.0)
Bisexual	10(1.9)
Unknown	68(13.1)
Year of plasma collection	
1991-2004	30(5.8)
2005-2009	491(94.2)
HIV-RNA load mean(SD), log copies/ml	4.54(0.75)

### Genetic subtypes

**Fig. 1:**
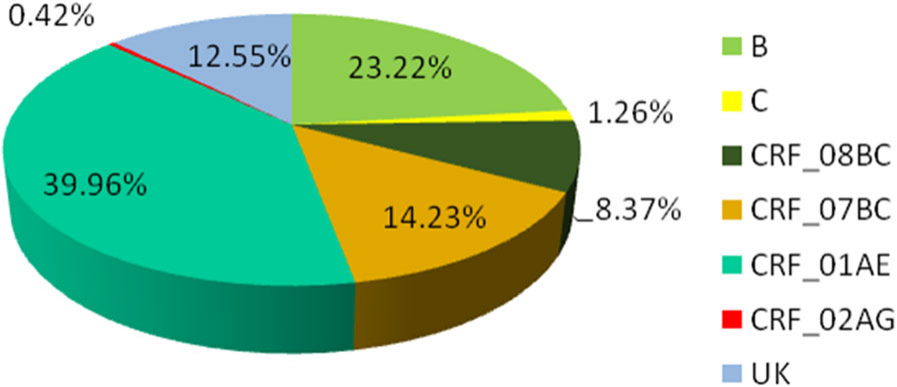
Distribution of HIV-1 subtypes and recombinants. The area of every section indicates the proportion of the subtypes and recombinants. UK: unknown

Based on RT-PCR and sequencing results, 478 (478/521) samples were amplified and sequenced successfully (GenBank accession numbers KU050197 - KU050674). A neighbor-joining tree of isolates in our program was constructed to determine subtype of each isolate and sequence relatedness. Subtyping assessment results revealed that substantial proportions of the study samples were CRF01_AE (40.0 %, 191/478) and subtype B (23.2 %, 111/478). Other subtypes included CRF07_BC (14.2 %, 68/478), CRF08_BC (8.4 %, 40/478) and subtype C (1.3 %, 6/478). Two (0.42 %, 2/478) CRF02_AG and one (0.21 %, 1/478) CRF06_CPX were also found. Figure 1 shows the distribution of the subtypes. Of these patients who were infected by sexual contact, most were CRF01_AE (44.2 %, 165/373), subtype B (16.4 %, 61/373) and CRF_07BC (16.1 %, 60/373); whereas the most common subtype among patients exposed to HIV-infected blood was subtype B (74.4 %, 32/43; Table 2).

**Table 2:** Prevalence of subtype among HIV_1+ patients infected by different route of transmissions

Route of transmission	B	C	CRF_01AE	CRF_07BC	CRF_08BC	Other	Total
Sexual contact	61(16.4)	3(0.1)	165(44.2)	60(16.1)	36 (9.7)	48(12.9)	373
Blood	32(74.4)	0(0.0)	2(4.7)	1(2.3)	2(4.7)	6(14.0)	43
Other	18(29.0)	3(4.8)	24(38.7)	7(29.2)	2(3.2)	8(12.9)	62
Total	111(23.2)	6(1.3)	191(10.0)	68(14.2)	40(8.4)	62(13.0)	478

### Resistance analysis

**Fig. 2:**
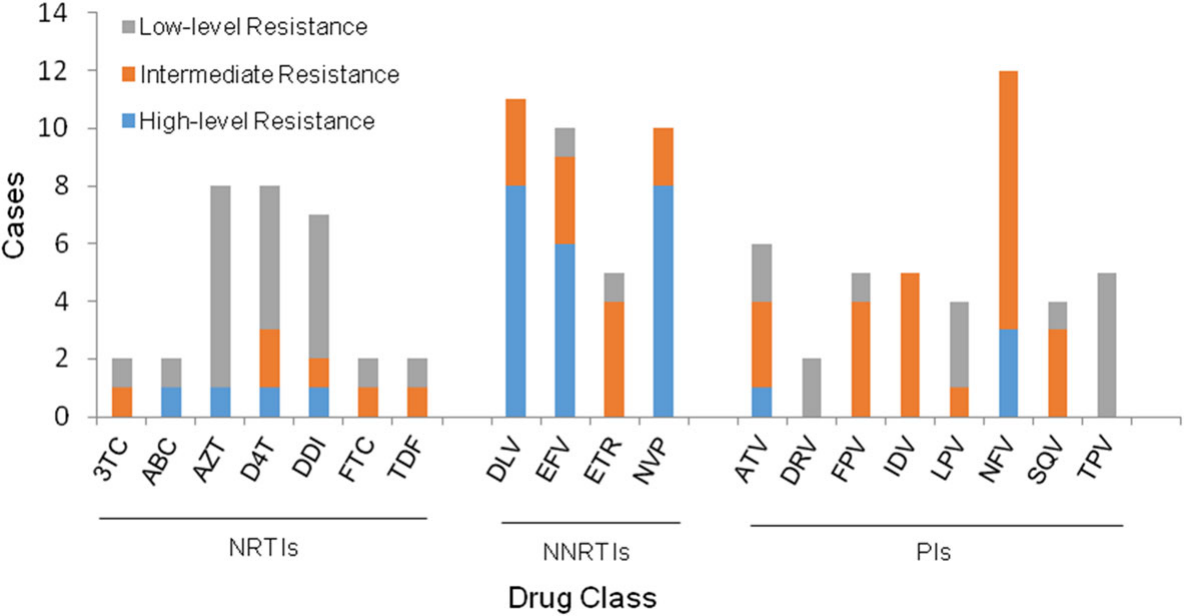
Resistance to different antiviral drugs in HIV-1-infected, antiretroviral therapy-naïve patients. The columns represent the various drugs and the column height indicates the number of the cases

All of the fragment sequences were translated and compared with the consensus B sequence in the Stanford University Network HIV-1 database [25]. A total of 32 (6.7 %) of 478 cases were found to harbor HIV-1 strains with at least one major drug-resistant mutation conferred by nucleoside reverse transcriptase inhibitors (NRTIs), nonnucleoside reverse transcriptase inhibitors (NNRTIs), or protease inhibitors (PIs), of whom 28 patients had mutations associated with reduced susceptibility to one ARV drug (Fig. 2). There was no significant difference (*p* > 0.05) in the prevalence of drug-resistant mutations among NRTIs (12/32), NNRTIs (11/32) and PIs (13/32). In addition, a substantial proportion of cases (9/11, 81.8 %) were highly resistant to NNRTIs, while only one case (1/12, 8.3 %), and 3 cases (3/13, 23.1 %) were highly resistant to NRTIs and PIs respectively, with a significant difference (*p* = 0.0050). The most commonly observed NNRTI resistance associated mutations were K103N (15.6 %, 5/32) and Y181C (12.5 %, 4/32). Of 13 cases resistant to PIs, 12 cases were resistant to Nelfinavir (NFV) resistance.

Comparison of the resistant rate in two major risk groups revealed that there was no significant difference (*p* > 0.05) between sexually infected individuals (28/373, 7.5 %) and patients infected by blood transfusions (3/43, 7.0 %). In addition, we did not find a significant difference in resistance in patients infected through heterosexual contact *versus* homosexual contact (*p* > 0.05). The prevalence of resistance in male and female was 8.1 % and 2.7 %, respectively (*p* > 0.05). Shenzhen had the highest rate of drug resistance at 11.6 %, and there was no significant difference (*p* > 0.05) among these regions (Table 3). There were different prevalence of resistance among subtype B, CRF_01AE and CRF_07BC/08BC (*p* = 0.0037). The susceptibility of CRF01_AE was significantly higher than subtype B (*p* = 0.0030).

**Table 3:** Drug resistance by demographic and clinical characteristics

Character	Number of patients who were successfully sequenced	Number of genotypic drug resistant patients (%)
Gender		
Male	345	28(8.1)
Female	110	3(2.7)
Unknown	23	1(0.2)
Area of origin		
Beijing	108	11(10.2)
Shanghai	74	3(4.1)
Guangzhou	48	2(4.2)
Shenzhen	43	5(11.6)
Fuzhou	51	4(7.8)
Henan	54	3(5.6)
Yunnan	74	2(2.7)
Xi’an	26	2(7.7)
Route of transmission		
Men who have sex with men	174	17(9.8)
Heterosexual contact	189	10(5.3)
Bisexual	10	1(10.0)
Blood	43	3(7.0)
Unknown	62	1(1.6)
Year of plasma collection		
1991-2004	28	2(7.1)
2005-2009	450	30(6.7)

**Fig. 3:**
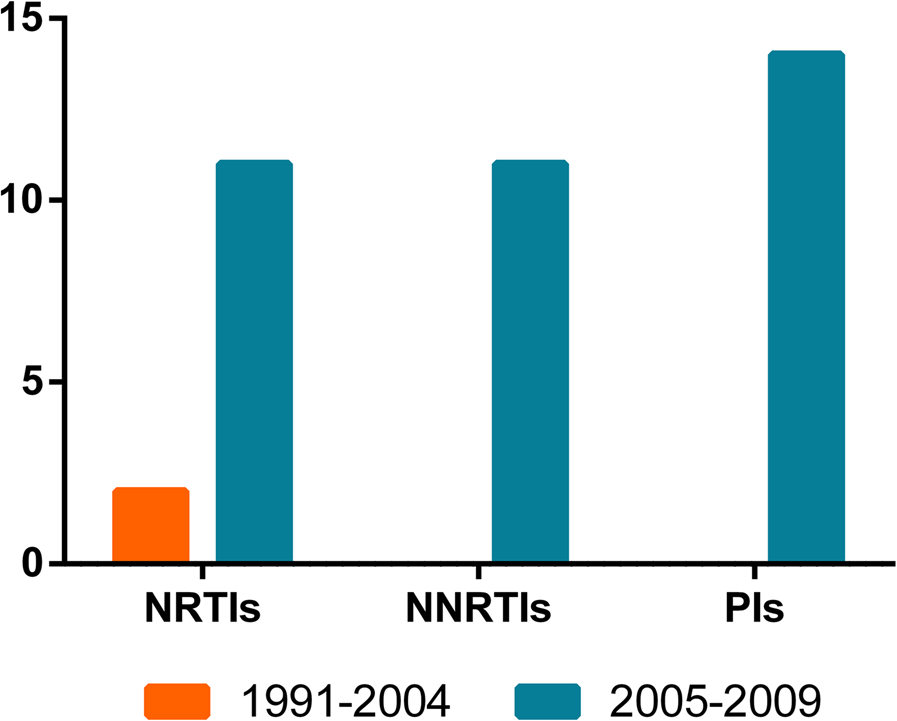
The time-dependent trend in any class of TDR. The orange columns represent the TDR in 1991-2004, and the blue columns represent that in 2005-2009. The column height indicates the number of the cases

As a result of the introduction of free ART in 2004, we compared the resistance between the two periods. No significant difference (*p* > 0.05) on resistance was observed in the 1991–2004 (2/28, 7.1 %) period and 2005–2009 (30/450, 6.7 %) period. And there is also no difference in TDR prevalence between the two periods in each origins (*p* > 0.05). Additionally, the differences on resistance to NRTIs, NNRTIs and PIs between the two periods were not statistically significant (*p* > 0.05, Fig. 3).

## Discussion

We report here the drug susceptibility of 521 treatment-naïve HIV-1-infected individuals at the Peking Union Medical College Hospital from 1991 to 2009. Four hundred and seventy eight gag-pol gene sequences (478/521) were sequenced successfully and the results of subtype and genetic resistance were obtained.

Various HIV-1 strains have been identified in China, of which CRF07_BC, CRF01_AE, CRF08_BC, and B/B’are the most common. Other reported strains include subtype G, subtype C, CRF55_01B and numerous unique recombinant forms (URFs) [26, 27]. Various HIV-1 subtypes/CRFs have been disseminated among different high risk populations. Our findings found that the most common subtype was CRF01_AE among the patients who were infected by sexual contact, and subtype B was found to circulate mainly in the blood-borne transmission population, which were consistent with previous Chinese studies [28, 29]. In the present study, we found that the main route of transmission remained blood-born route for the subjects in Henan province, of which subtype B was dominant, as a result of HIV infection outbreak in the mid-1990s in paid blood donors in Henan.

Our studies determined that the TDR prevalence in China was 6.7 %, a level classified as “intermediate” according to the WHO thresholds, and which was slightly lower than that observed in some industrialized countries. In the present study, we found no significant differences in the presence of TDR by demographic characteristic, which was similar with a previous study [8]. Here we showed that there was no significant difference in the prevalence of drug-resistant mutations among the three antiretroviral classes (PI, 2.7 %; NRTI, 2.5 %; and NNRTI, 2.3 %). In addition, we noted that the prevalence of HIV-1 strains with high level of resistance mutations to NNRTIs were significantly higher than those to NRTIs and PIs (*p* < 0.0050). The higher prevalence may be related to mutations associated with decreased susceptibility to NNRTIs with a low genetic barrier, which are rapidly generated and emerge early in the selection process [30].

It is known that there is an approximate 8–10 % genetic diversity exists in pol gene among various subtypes; therefore drug resistance susceptibility of genotype virus may be different. Here we showed a strong association between the prevalence of TDR and subtypes. There was different prevalence of resistance among subtype B, CRF_01AE and CRF_07BC/08BC. Resistance was significantly higher in ARV-naïve patients who were infected with subtype B than in those infected with CRF01_AE viruses (*p* = 0.0030), which was similar with previously published studies [17–19, 31, 32]. Although subtypes may vary in mutational pathways, it is unclear whether subtype B was more prone to mutation [33]. As the predominant prevalence in China, HIV-1 subtype B viruses have been exposed to ARV drugs for a longer period than other subtypes [26, 34].

This study was performed on ART-naïve individuals. It has been reported that resistance testing prior to the initiation of treatment in untreated populations with HIV infection is cost-effective and may be beneficial to the patient [35, 36]. It is commonly considered that resistant HIV-1 variants in ART-naïve patients are transmitted from ART-experienced patients or other ART-naïve individuals with drug-resistant strains. There are some studies indicating that drug resistant mutations among treatment-naïve patients were associated with ART used prior to and early in the HAART era [16]. As the ART use has been extended in China in recent years, it becomes increasingly important to monitor HIV-1 genetic diversity and TDR. Hence, we also tested whether the free ART program, which was implemented in 2003, has influenced the prevalence of overall TDR among ART-naïve patients in China. It should be noted that contrary to the reports from other countries that have implemented broad-access programs to ART [37, 38], results of our analysis showed that there was no time-dependent trend in the overall TDR, origins, or in any class of drug resistance. Although we did not observe evidence of an increasing prevalence of TDR over years in the sampling patients, the number of HIV-1-infected individuals in our study may be small.

While we did not study a random sample of HIV-1-infected individuals, this was a large, diverse population with a wide time span from a widespread referral network in China, and our study well reflects the time trend and demographic characteristics of TDR.

## Conclusions

In summary, in this study, we fulfilled the comprehensive investigation on the circulation of TDR of three ARV classes in China. The overall prevalence of TDR remains intermediate in ART-naïve individuals, and has a stable TDR time trend. These findings enhance our understanding of HIV-1 drug resistance prevalence and time trend, and provide some guidelines for proposing efficacious and effective programs to prevent transmission of HIV-1drug resistant strains.

## Abbreviations

ART: Antiretroviral therapy
ARV: Antiretroviral
CRF: Circulating recombinant form
HIV: Human immunodeficiency virus
NNRTIs: Nonnucleoside reverse transcriptase inhibitors
NRTIs: Nucleoside reverse transcriptase inhibitors
PIs: Protease inhibitors
TDR: Transmitted drug resistance
URF: Unique recombinant form
